# Supplementary material for: A Cross-Sectional Study: Structural and Related Functional Connectivity Changes in the Brain: Stigmata of Adverse Parenting in Patients with Major Depressive Disorder?
Source: Brain Sci. 2023 Apr 21;13(4):694. doi: 10.3390/brainsci13040694 (PMC10136887; doi:10.3390/brainsci13040694)
Supplement: Supplementary file 1 [file brainsci-13-00694-s001.zip › brainsci-2323461-supplementary.pdf]

Supplementary Material

S1. Cluster analysis

We selected 4 variables (maternal Warmth factor score, maternal Authoritarianism factor score, paternal Warmth factor score and paternal Authoritarianism factor score) of 125 participants with which we performed the Ward minimum-variance hierarchical clustering using a Euclidean distance metric with complete linkage. The analysis was conducted in IBM SPSS Statistics v25.0 (IBM Statistics, Armonk, NY, USA).

S2. Sensitivity analyses

In order to increase the robustness of FC results, we performed a sensitive analysis as following, extracting a sphere with a radius of 6 mm and centered on coordinates x = 30, y = -67.5, z = 55.5 as the region of interest 2 (ROI 2) using Wake Forest University (WFU) Pickatlas 3.0.5b [1]. And we found a significant increased FC in the left medial superior frontal cortex(smFC) in AP group which is the same as the previous outcome (see **Figure S1**).

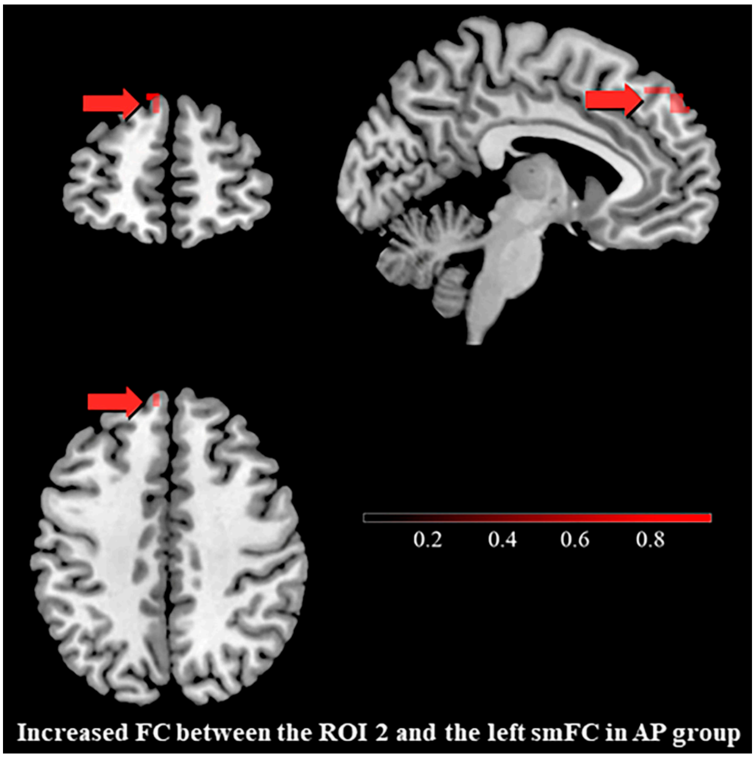

Figure S1: FC between the ROI 2 and the left smFC also increased in AP group (22 voxels, $T = 4.242$, $p < 0.001$, GRF correction).

S3. Exploratory Data Analysis

S3.1 The relationship between changes of FC and specific parenting style

In the DMN, voxel-wise FC between the right SPL and the right smFC (Cluster 1), the right smFC (Cluster 2) and the left smFC (Cluster 3) were significantly different between AP group and OP group. After the sensitivity analyses, we thought the result in Cluster 3 was more robust, so we defined Cluster 3 as ROI 3. And in the DMN, the voxel-wised FC between the ROI 3 and the right middle temporal gyrus (Cluster 4) were significantly different between the two groups.

To further explore the relationship between changes of FC and specific parenting style, we performed Pearson correlation. We extracted the FC signals of Cluster 1, Cluster 2, Cluster 3 and Cluster 4 separately and calculated the Pearson correlation between them and PBI item scores. As shown in Table 1 and Figure 2, we found the FC signals between the right SPL region and Cluster 1, Cluster 2, Cluster 3 were negatively correlated with parental warmth scores (r = -0.368 ~ -0.207, $p < 0.05$). The FC signal between the left smFC and the Cluster 4 are negatively correlated with maternal warmth scores (r = -0.504, $p < 0.001$) and paternal warmth scores (r = -0.219, $p < 0.05$) while positively correlated with maternal authoritarianism scores (r = 0.251, $p < 0.001$).

Table S1. Pearson correlation between PBI item scores and FC signals.

	Maternal Warmth	Maternal Authoritarianism	Paternal Warmth	Paternal Authoritarianism
FC signal of Cluster 1	-0.368***	0.139	-0.232*	0.010
FC signal of Cluster 2	-0.264**	0.021	-0.255**	0.047
FC signal of Cluster 3	-0.368***	0.033	-0.207*	0.013
FC signal of Cluster 4	-0.504***	0.251**	-0.219*	0.145

$^{*}p < 0.05$, $^{**}p < 0.01$, $^{***}p < 0.001$ (two-tailed)

Cluster 1, Cluster 2 and Cluster 3 are the significantly different regions in which that the voxel-wised FC between the right SPL and the DMN. Cluster 4 is the significantly different region in which that the voxel-wised FC between the ROI 3 and the DMN.

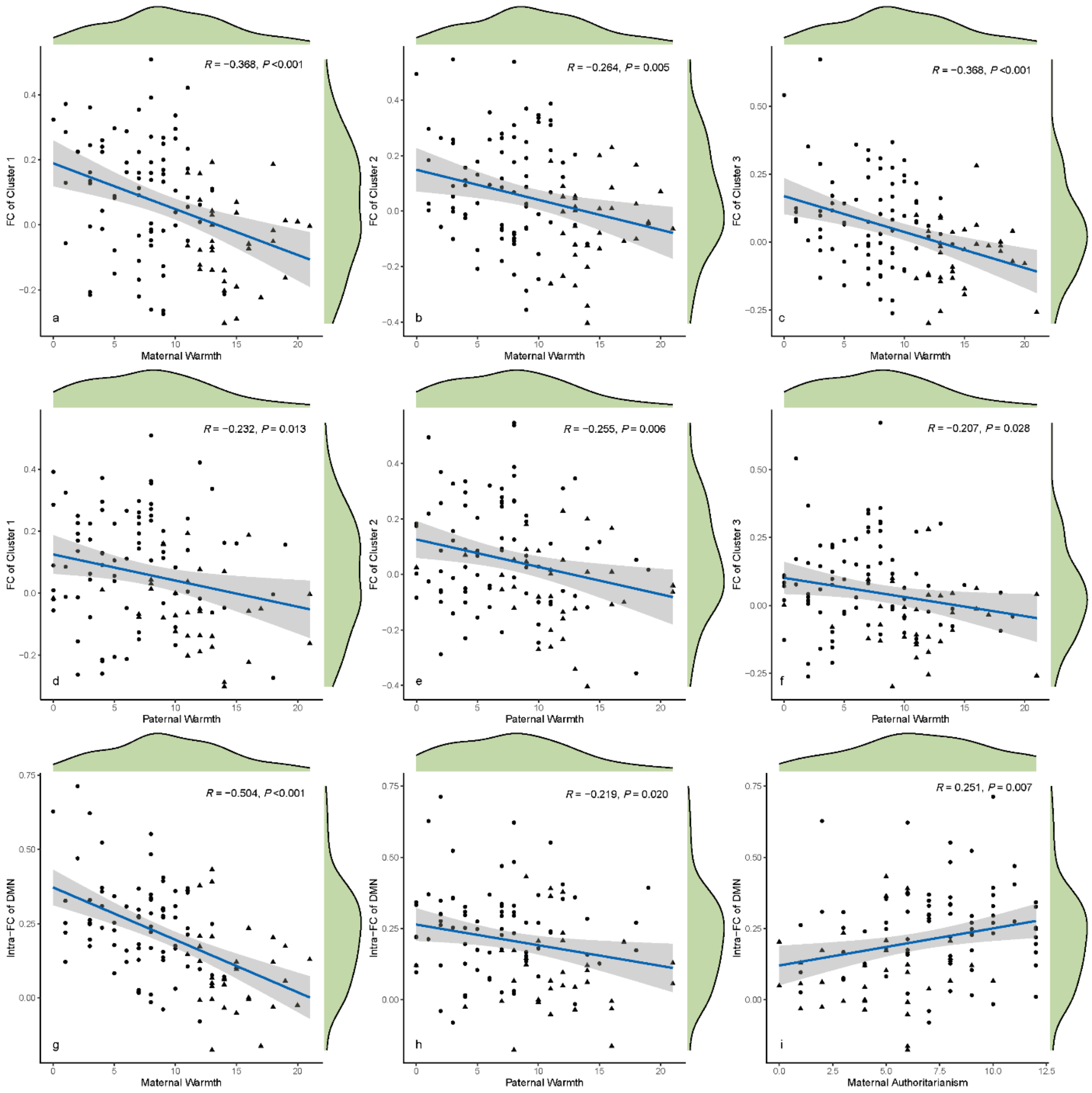

Figure S2: FC signals between the right SPL region and Cluster 1 were negatively correlated with parental warmth scores (Figure S2a and S2d). FC signals between the right SPL region and Cluster 2 were negatively correlated with parental warmth scores (Figure S2b and S2e). FC signals between the right SPL region and Cluster 3 were negatively correlated with parental warmth scores (Figure S2c and S2f). The FC signal between the left smFC and the Cluster 4 are negatively correlated with maternal warmth scores (Figure S2g and S2h). The FC signal between the left smFC and the Cluster 4 are positively correlated with maternal authoritarianism scores (Figure S2i). The triangle represents the AP group and the circle represents the OP group.

S3.2 Correlations between PBI item scores and depressive symptoms

Although we failed to find significant group differences between the AP and OP groups by *t*-test, to further explore the relationships between parenting style and depressive symptoms. We calculated Pearson's correlation coefficients between scores of PBI items and scores of anxiety/somatization factor, sleep disturbance factor, psychomotor retardation factor from HAMD-17 and SHAPS. Only a significant positive correlation was found between paternal Authoritarianism and anxiety/somatization, but trends reflected by almost all correlation coefficients were consistent with our expectations. Degree of severity of depressive symptoms such as anhedonia, sleep disturbance, psychomotor retardation and anxiety/somatization is positively correlated to scores of parental authoritarianism, negatively to scores of parental Warm (see **Table S2**).

Table S2. Correlation between item scores of PBI and total score of the SHAPS and item scores of the HAMD-17.

Scales	Maternal Warmth	Maternal Authoritarianism	Paternal Warmth	Paternal Authoritarianism
SHAPS	0.095	-0.016	0.070	-0.030
Anxiety/somatization	-0.082	0.152	-0.076	0.218*
Sleep disturbance	-0.035	0.011	-0.096	0.125
Psychomotor retardation	-0.015	-0.014	-0.011	0.136

* Correlation is significant at the 0.05 level (two-tailed).

References

1. Maldjian, J.A.; Laurienti, P.J.; Kraft, R.A.; Burdette, J.H. An automated method for neuroanatomic and cytoarchitectonic atlas-based interrogation of fMRI data sets. *Neuroimage* **2003**, *19*, 1233-1239, doi:10.1016/s1053-8119(03)00169-1.
